# Supplementary material for: Reinforcing materials modelling by encoding the structures of defects in crystalline solids into distortion scores
Source: Nat Commun. 2020 Sep 17;11:4691. doi: 10.1038/s41467-020-18282-2 (PMC7499431; doi:10.1038/s41467-020-18282-2)
Supplement: Supplementary file 1 — Supplementary Information [file 41467_2020_18282_MOESM1_ESM.pdf]

SUPPLEMENTARY MATERIALS

Reinforcing materials modelling by encoding the structures of defects in crystalline solids into distortion scores

Alexandra M. Goryaeva et al.

CONTENTS

I. SUPPLEMENTARY NOTE 1: Comparison of MCD and OCSVM to provide a distortion score of LAE 3

Correlation with local atomic energy 3

Stratification of defect structures 4

II. SUPPLEMENTARY NOTE 2: Multidimensional distortion score for analysis of displacement cascades and detection of targeted defect types 6

Construction of the training data set with multimodal distribution of LAEs 6

Analysis of the displacement cascade calculations using a multidimensional distortion score 9

III. SUPPLEMENTARY NOTE 3: Energy profile of the screw dislocations in bcc iron 11

Stratification of the bulk structure 11

Reconstruction of the Peierls barrier from mean force calculations in a big simulation cell 14

IV. SUPPLEMENTARY NOTE 4: Performance of the GAP potential for modelling radiation induced defects in bcc iron 16

Self-interstitial atoms and their clusters 16

Vacancies and their clusters 20

Methods 20

Supplementary References 21

I. SUPPLEMENTARY NOTE 1: COMPARISON OF MCD AND OCSVM TO PROVIDE A DISTORTION SCORE OF LAE

In this work we are interested in the outlier detection methods with a smooth decision function that can provide a measure, appropriate to describe a distortion score of local atomic environments (LAEs). Here we compare the performance of two ML methods, Minimum Covariance Determinant (MCD) [13, 14, 25] and One Class Support Vector Machine (OCSVM) [29, 30]. Both methods exhibit an excellent performance to distinguish between atoms-inliers (known LAEs) and atoms-outliers (unknown or defect-related LAEs). In this section, we report the performance of linear OCSVM to provide an accurate measure of LAEs via the distortion score based on the distances d_{SVM} (Eq. 8 in the main text of the paper) and compare it with robust distance d_{RB} (Eq. 5 in the main text of the paper).

SVM is a widely used ML technique for the tasks of classification, regression, and outlier detection. Binary classification based on linear SVM was previously shown to be efficient for the analysis of atomic dynamics in disordered solids [8, 27, 28] and at grain boundaries in polycrystals [31]. In the framework of SVM, the distortion score stands for the distance d_{SVM} from the hyperplane that separates inliers from outliers (see Section One Class Support Vector Machine in the Methods of the main text). SVM distances have a positive sign for inliers and a negative sign for the outliers.

In contrast to SVM, MCD is a less commonly used technique, aimed exclusively at outlier detection. To the best of our knowledge, MCD has never been applied for the needs of atomistic materials science. The distortion score from MCD corresponds to the robust distance d_{RB} (see Section Minimum Covariance Determinant in the Methods of the main text), which describes the distance from the centre of the cloud of the defect-free bulk structures.

Correlation with local atomic energy

Here, we examine the correlation of SVM distances d_{SVM} with local atomic energies. Figure 1 shows the best correlation of d_{SVM} with the local atomic energies based on the same structural data as reported in Figure 2 in the main text of the paper. Overall, linear SVM distances are positively correlated with the local atomic energies, however, the correlation coefficient R^2 is systematically lower than 0.7, which does not allow to use d_{SVM} as an accurate measure of LAEs. Thus, compared to the SVM method (Fig. 1), a straightforward application of robust MCD provides better a correlation with local atomic energies (Figs. 2 and 9 in the main text).

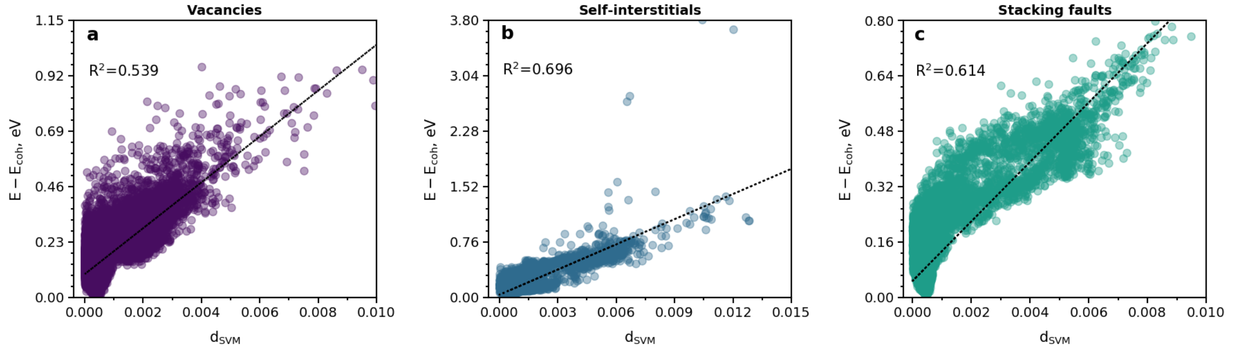

Supplementary Figure 1: Correlation of the local energy from GAP interatomic potential for Fe with the magnitude of d_{SVM} distances obtained using linear OCSVM with ν=0.005. Each point on the plot represents an individual atomic environment. The structural data is taken from the GAP potential database [9] and fully corresponds to that from Figure 2 in the main text of the manuscript.

Previous study by Sharp et al. [31] used d_{SVM} to estimate the so-called softness of atoms, a continuous, signed, scalar measure, which was used to describe the likelihood of atoms to rearrange under thermal fluctuations within the grain boundaries in fcc Al. Similarly to our study, Ref. [31] reported a positive correlation of SVM distances with local atomic energies with the large spread d_{SVM} for a given energy value. Thus, while using completely different formalisms to compute local atomic energies (ML GAP potential [9] vs conventional EAM potential [22]), different atomic descriptors (bispectrum SO4 [3] vs G_2 and G_3 functions [4]) and different training databases to describe defects in different materials (bcc Fe vs fcc Al), our calculations are consistent with those by Sharp et al. [31] and indicate that d_{SVM} is not directly attributable to the local energy.

Using SVM with a non-linear kernel could help improving the correlation, however, it will lead to a drastic increase of numerical cost of the analysis (learning process on the database with M LAEs requires M^3 numerical operations) and will imply a careful choice of the kernel form and parameterization. Moreover, the chosen kernel can not remain universal for different structural types and the parameterization should be adjusted by the user for the given application. This characteristics of the kernel-based methods do not allow to consider them as a universal method of structural analysis, suitable for the Applications 1 and 2 described in the main text of the paper.

Stratification of defect structures

Here we examine the correlation between the SVM distances with those from robust MCD (Fig. 2) for the self-interstitial (SIA) clusters in bcc Fe. For the simple $I_1^{\langle 110\rangle}$ dumbbell (Fig. 2a), the

two techniques perform similarly. The pair of atoms that form a dumbbell (yellow points) are characterised by large SVM and MCD distortion scores and can be unambiguously distinguished from the rest of atomic array. For more complex structures of 3D interstitial clusters (Fig. 2b, c), the two techniques exhibit a different performance for the outliers with large distances. For the atoms that contribute to the defect structure itself (green and yellow points in Figure 2b, c), SVM distances saturate. Thus, for the Gao-triangle I_2^{NP} (Fig. 2b), SVM provides only one possibility to localise the interstitial atoms, while MCD enables a clear distinction between the two families of atoms (structure inserts A and B) that contribute to the defect structure. In the case of the C15 cluster, it is not possible to isolate the I_4^{C15} polyhedra based solely on the SVM distances (Fig. 2c, structure insert B). For the same structure, MCD provides a possibility for a more thorough analysis, which correctly identifies the shell of the C15 polyhedra (Fig. 2c, structure insert B) and the atoms centring them (Fig. 2c, structure insert C with yellow atoms). These examples illustrate that defect stratification based on the distortion score d_{RB} provided by robust MCD is better suited for detailed analysis of complex defect structures with well pronounced outlier atoms.

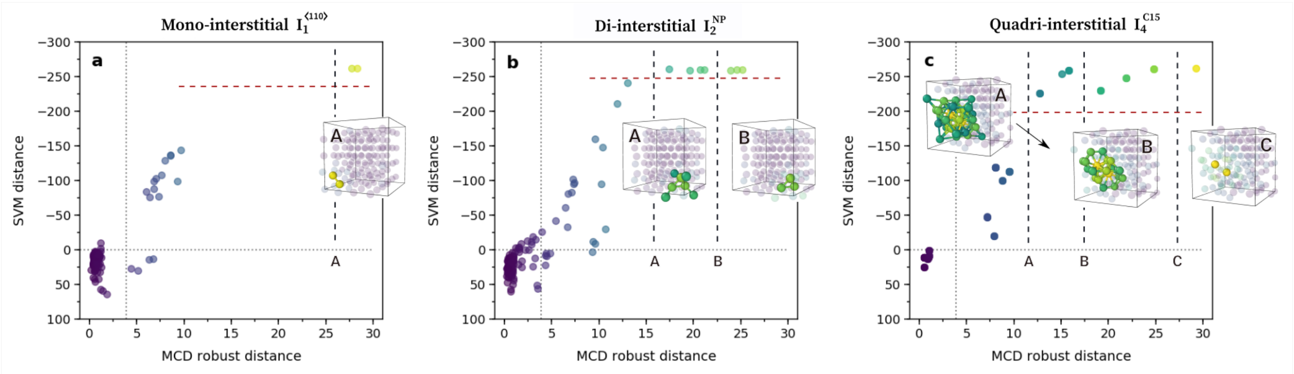

Supplementary Figure 2: SVM vs robust MCD distances in the atomic arrays with different types of self-interstitial: (a) $I_1^{\langle 110 \rangle}$ dumbbell; (b) I_2^{NP} Gao-triangle; (c) I_4^{C15} cluster. Each point on the plot represents an individual atom in a simulation box. The points and atoms are coloured according to the MCD robust distance. The grey dotted lines indicate the critical SVM and robust MCD distances between the bcc bulk and the defect. The horizontal dashed red lines indicate the SVM cutoff distances that allows to isolate interstitial atoms; the vertical dashed black lines (labelled as A, B, C) indicate several robust MCD distances that can be applied to stratify the defect structures. The insert structures A, B, C depict the structures of defects, obtained after applying the corresponding stratification based on MCD distances. The defect structures obtained using SVM systematically correspond to the insert structures A in the (a,b,c) subplots.

II. SUPPLEMENTARY NOTE 2: MULTIDIMENSIONAL DISTORTION SCORE FOR ANALYSIS OF DISPLACEMENT CASCADES AND DETECTION OF TARGETED DEFECT TYPES

Under irradiation by high energy ions or neutrons, crystalline solids can be supersaturated with defects, i.e., the defects in irradiated materials are present at concentrations well above the thermodynamic equilibrium. The effects of radiation on the material's properties and ageing depend on a combination of multiple factors, such as the type of induced radiation, the nature of irradiated material and the energy of the radiation particles. Once irradiated, the primary knock-on atom (PKA) induces a chain of collisions, leading to a displacement cascade. During this collision phase, atomic displacements mainly occur in the form of point defects, e.g., self-interstitial atoms (SIAs), vacancies, Frenkel pairs. These defects may eventually recombine and annihilate or regroup and form larger defects like 2D and 3D clusters, dislocation loops, stacking faults, etc. The morphology of primary defects, i.e., those which were formed as a direct consequence of the cascade, defines their mobility and stability with respect to each other. These factors play an an important role in subsequent micro-structural evolution in the material.

Molecular dynamics calculations of displacement cascades provide the essential physical basis for understanding of atomic-scale processes that occur during primary displacement events [7, 26, 36]. However, interpretation of such calculations often implies a challenge [5, 23] due to the complex structural processes that produce a rich variety of defects, as well as due to the large size of the simulation cells, which can reach up to few tens of millions of atoms when computing the damage produced by high energy PKAs (e.g., 200 keV and more).

Here we demonstrate the performance of MCD for analysis of displacement cascades in fcc Cu and, in particular, for detection of targeted defect types in the damaged structure using a multidimensional distortion score.

Construction of the training data set with multimodal distribution of LAEs

The distortion score provided by robust MCD is adapted for training data with unimodal distribution. However, the complexity of structural data selected as a reference for training not always can be represented by one Gaussian. Below we demonstrate how this limitation for construction of training data sets suitable for MCD analysis can be overcome using a modal decomposition of the database. As a possible solution, the multimodal training database $\mathcal{D}$ can be decomposed in several unimodal data sets $\mathcal{D}_1 \oplus \mathcal{D}_2 \oplus \ldots \oplus \mathcal{D}_n$. After such a decomposition, a robust distance can

be computed with respect to each unimodal dataset $\mathcal{D}_i$, forming thus a multidimensional distortion score. The multidimensional distortion score is particularly suitable for advanced structural characterization of any and all defects that are simultaneously present in a material. When using a multidimensional score, several measures can be associated to each atomic environment in such a way that each component of the distortion score is related to a specific reference structure with unimodal distribution of LAEs in the descriptor space. Here we aim to construct a training data set for detection of various interstitial-type defects in fcc Cu.

Choosing relevant structures for the training data set

Fcc metals are generally known to have small interstitial defects, like $\langle 100 \rangle$ dumbbells, alone or packed into 3D structures, like those described by Ingle et al. [15]. The larger defects are 2D and represented by faulted (Frank) $\frac{1}{3}\langle 111 \rangle\{111\}$ or unfaulted (prismatic, perfect) $\frac{1}{2}\langle 110 \rangle\{111\}$ dislocation loops. The fault inside the $\frac{1}{3}\langle 111 \rangle\{111\}$ loop has the hcp structure. Consequently, the interstitial-type defects of interest can be described here using a multidimensional distortion score where each dimension is spanned by the robust MCD distance associated to the following structural classes: fcc, hcp, $\langle 100 \rangle$ dumbbells and 3D SIA clusters.

Training structures and their representation in the descriptor space

The training structures are created using $a_0^{fcc} = 3.615$ Å for the fcc bulk and for the $\langle 100 \rangle$ and SIAs embedded in fcc bulk. The hcp reference structures are created using $a_0^{hcp} = a_0^{fcc}/\sqrt{2}$ and $c_0^{hcp}/a_0^{hcp} = \sqrt{8/3}$. On the atomic positions of the perfect structures was applied zero mean white noise with normal distribution $\mathcal{N}(0, \sigma = 0.08$ Å$)$. Including the configurations with noise into the training data set allows to increase sensitivity of the model to perturbations such as thermal noise or structural relaxation around defects. For the distortion score components associated with the fcc and hcp bulk structures, the MCD model is trained on $M = 15{,}000$ atomic environments for each structural type. For the model training related to the $\langle 100 \rangle$ dumbbells and 3D SIA clusters, we employ exclusively the LAEs that form the corresponding defects (e.g., exclusively the atoms forming the $\langle 100 \rangle$ SIA dumbbells). The training data set for detection of $\langle 100 \rangle$ and the 3D clusters consists of 1,000 and 1,840 atomic environments, respectively. The training and test structural data are represented in the feature space of bispectrum SO(4) [3], with cutoff distance $R_c = 4.6$ Å and angular moment $j_{max} = 3.5$, resulting in 40 descriptor components for each atomic environment.

Analysis of the multimodal distribution of the training data

Once the relevant structural classes are defined and the training data is represented in the descriptor space, we apply a Gaussian mixture model (GMM) [16, 19] to the training data represented by the

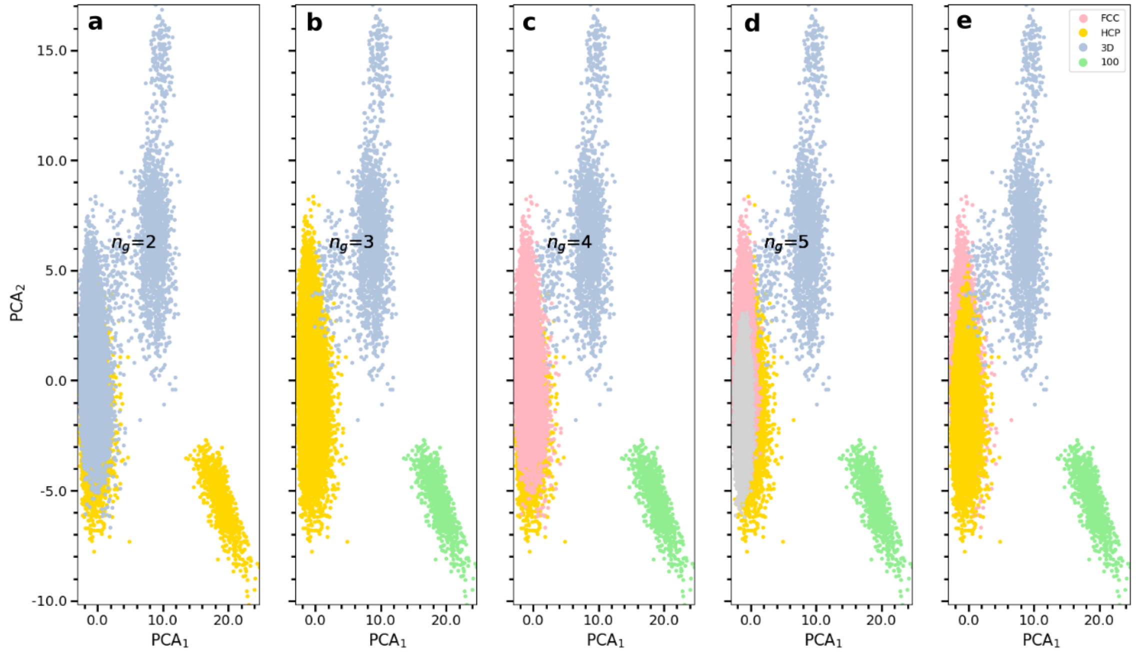

Supplementary Figure 3: The multimodal Bayesian Gaussian analysis [16, 19] of the fcc, hcp, ⟨100⟩ and 3D SIA training atomic environments. For better visualization, the 40-dimensional descriptor space of training data is represented in 2D using linear PCA dimensionality reduction. The subplots (a-d) illustrate the GMM analysis using from $n_g = 2$ up to $n_g = 5$ Gaussians (n_g denotes the number of Gaussians). Each point on the plot represent an atomic environment. The atomic environments belonging to the same Gaussian are depicted with the same colour. The subplot (e) shows the training configurations using the same PCA representation as for the GMM analysis (a-d), whilst the colours correspond to the structural type: the ⟨100⟩ dumbbells, 3D clusters, hcp and fcc are shown in green, blue, pink and yellow, respectively.

four selected structural classes. The mixture model based on Gaussians is particularly convenient for modelling continuous observations in the descriptors space, such as our training data. This data will be denoted as $\mathbf{x} \in \mathbb{R}^D$. Consequently, the data can be written as mixture of n_g Gaussians of dimension D of mean $\mathbf{m_i}$ and covariance matrix $\mathbf{S}_i$:

$$p(\mathbf{x}) = \sum_{i=1,n_g} p(\mathbf{x}|\mathbf{m}_i, \mathbf{S}_i)p_i, \quad (1)$$

where p_i is the mixture weight for component i. The optimal set parameters $\mathbf{m}_i, \mathbf{S}_i, p_i$ can be obtained using standard maximum likelihood optimization. This procedure is applied simultaneously for the four training classes that were identified as necessary for the structural analysis of the displacements cascade.

The GMM analysis using from $n_g = 2$ to $n_g = 5$ Gaussians is presented in the Fig. 3a-d. The 40-

dimensional training data is visualised in 2D after a linear PCA decomposition. The GMM analysis indicates that $n_g = 4$ (Fig. 3c) accurately explains the distribution of the training data. In this case, the number of Gaussians (Fig. 3c) is equal to the number of *physical* classes of the reference structures (Fig. 3e). This conjecture is supported by the fact that all atomic environments of the reference structures were generated using a Gaussian distribution of atomic displacements.

The GMM analysis with 5 Gaussians (Fig. 3d) emphasises the presence of a bi-modal distribution within the hcp structures. This comes from the fact that the training hcp structures with noise do not represent a perfect 2-layer AB close-packed structure and that A and B layers are not strictly identical from the point of view of symmetry. Computing a distance from each of the hcp Gaussians will mean distinguishing between A and B layers within the hcp structure, which is not the aim of this analysis. The aim is to detect the local regions with hcp lattice within the fcc lattice. Consequently, taking into account that the training hcp LAEs are also well described by the unimodal distribution (Fig. 3c, e), in this particular case, it is sufficient to keep a single distance associated to the hcp structure and to perform the analysis of the displacement cascade using a 4D distortion score, which consists of d_{RB}^{fcc}, d_{RB}^{hcp}, $d_{RB}^{\langle 100 \rangle}$ and d_{RB}^{3D}.

Analysis of the displacement cascade calculations using a multidimensional distortion score

The analysis is performed for the fcc Cu simulation box with 5,324,000 atoms, taken from the dataset R093 [23] of the open-source database cascadesdb.org by the International Atomic Energy Agency (IAEA). Similarly to the procedure described in the main text of the article (see maint text, Fig. 1), the structural damage can be isolated from the defect-free bulk structure using the distortion score component related to the fcc Cu structure (Fig. 4a, c, e). In order to identify and localise the defects of interest in the cloud of structural damage, the other three components of the constructed 4D distortion score can be applied, namely, those related to hcp, $\langle 100 \rangle$ dumbbells and 3D SIA clusters. In this case, the corresponding component of the distortion score distortion score (robust MCD distance) will describe the proximity of LAEs in the analyzed system to the targeted defect. The LAEs with small MCD distances from the defect structures (inliers) will correspond to the defect of interest. The detected stacking fault structures and their aggregates are depicted in Figure 4b. The purple areas within this defects are identified as hcp structure. The atoms at the edges of the defects have higher hcp distortion scores with respect to the middle areas. The atoms at the edges are neighbouring with hcp faults, fcc bulk and sometimes with other defects, therefore there LAEs differ from the training hcp bulk structures. Figure 4d illustrates the detected $\langle 100 \rangle$

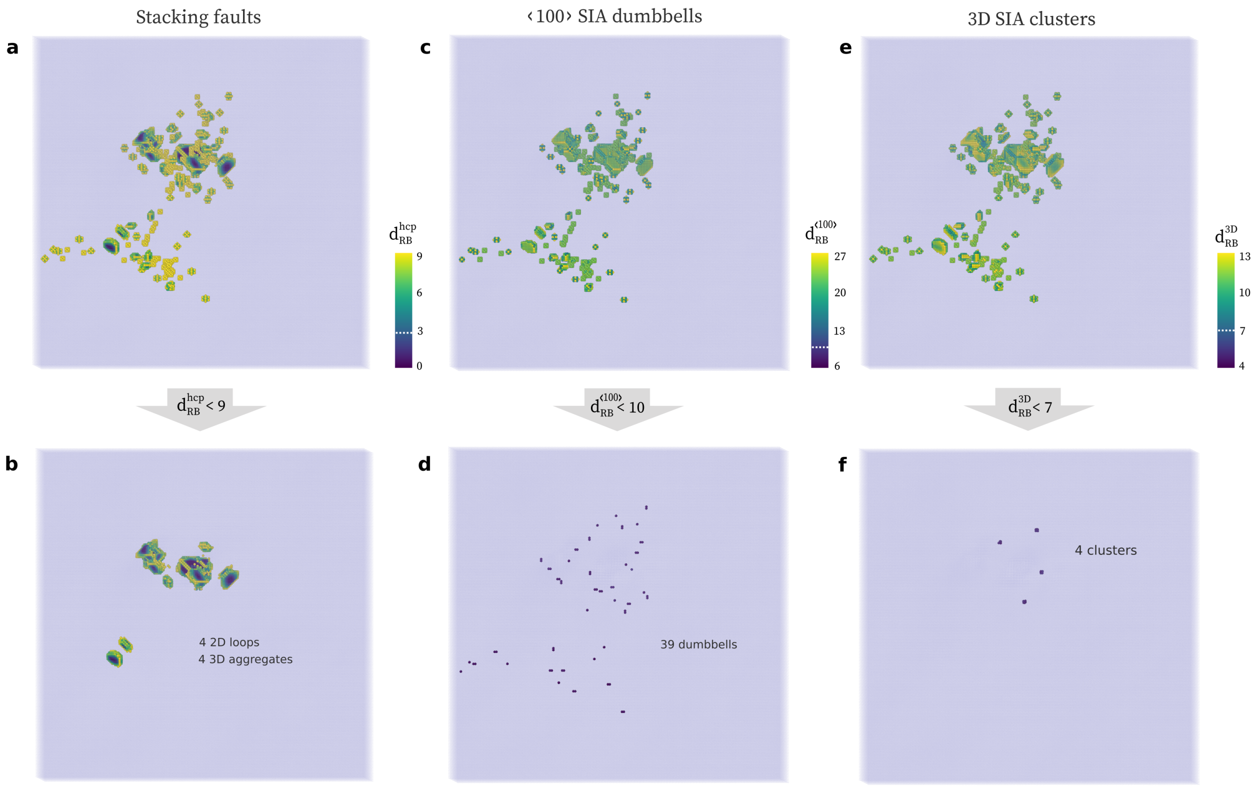

Supplementary Figure 4: Structural damage produced by 100 keV displacement cascade in fcc Cu. (a, c, e) The totality of damage produced by the displacement cascade. The atoms identified as fcc bulk are shown in transparent purple in all the subplots. The atomic environments identified as deviating from fcc Cu are coloured according to their distortion score (MCD robust distance) related to: (a, b) hcp bulk structure; (c, d) ⟨100⟩ SIA dumbbells; (e, f) 3D SIA clusters described by Ingle et al. [15]. (b, d, f) The detected defects from the three targeted classes: (b) the 2D hcp stacking faults and their 3D aggregates; (d) the ⟨100⟩ SIA dumbbells; (f) the 3D SIA clusters. The simulation box for the analysis is taken from open-source database cascadesdb.org by IAEA, dataset R093 [23] with 5,324,000 atoms.

SIA dumbbells that are concealed in Figure 4a. More interestingly, this method can be applied for detection of rare defects or such defect types that are challenging to identify using standard geometry based methods. Figure 4e illustrates the detected 3D SIA clusters, which were detected using the MCD model trained on LAEs of rare 3D clusters described by Ingle et al. [15].

Thus, using a multidimensional distortion score, it is possible to perform a detailed analysis of the systems with complex damage. Here, we have unambiguously localised the targeted defects, including the rare 3D SIA structures (Fig. 4e), which are difficult to identify with traditional techniques.

III. SUPPLEMENTARY NOTE 3: ENERGY PROFILE OF THE SCREW DISLOCATIONS IN BCC IRON

Stratification of the bulk structure

In Application 2 (main text of the article) we demonstrate how stratification of defects based on the distortion score of LAEs can serve for the reconstruction of migration energy barriers of defects from mean force calculations. In the context of this application, dislocations represent a particular type of defects as they create a long-range displacement field, which is not localised around the dislocation lines. The atoms-outliers are compactly located around the dislocation line and, in contrast to other defect types (e.g., Fig. 5a,b in the main text), integrating the forces over the outlier region is not sufficient for reconstruction of the Peierls barrier. In this case, accurate reconstruction of the barrier requires including the surrounding bulk atoms (inliers) that are most impacted by the elastic displacement field produced by the dislocations.

Figure 5 illustrates stratification of the simulation cells with $\frac{1}{2}\langle 111\rangle$ dislocation dipoles gliding in the $\{110\}$ plane in bcc Fe. The corresponding reconstruction of the Peierls barrier and atomic structures are presented in Fig. 5 c,d in the main text. As expected for the dislocations, considering only atoms-outliers with $d_{RB} > 2.9$ (the threshold provided by the MCD model indicated with red dashed line in Fig. 5) results in underestimated migration barrier (main text, Fig. 5c). In order to select the relevant bulk atoms, we consider possibilities for bulk stratification along the NEB path (Fig. 5b, d, f). Here, we mainly focus on the snapshots with the reaction coordinate $\zeta = 0$, $\zeta = 1$ (Fig. 5b), $\zeta = 0.26$, $\zeta = 0.74$ (Fig. 5d) and $\zeta = 0.5$ (Fig. 5f). These configurations correspond to the minimum- and maximum-energy points along the migration path (main text, Fig. 5c). The stratification levels B with $d_{RB} = 1.45$ and C with $d_{RB} = 1.0$ within the bulk structure (shown with dashed black lines in Fig. 5b,d,f) are selected in such a way that they separate distinct distortion score layers in all the selected snapshots. The analyzed simulation cells contain dislocations that are only distant by 17.45 Å, which imposes strong elastic interaction between the cores. In such small simulation cells, consideration of at least half of the bulk atoms is indispensable for reliable reconstruction of the migration barrier. In this particular case, integration of the forces over the region with $d_{RB} > 1.0$ is sufficient for reconstruction of the Peierls barrier with 95% accuracy. For larger simulation cell with less important interaction between the cores, the proportion of the relevant bulk atoms will be drastically reduced. The example of the Peierls barrier reconstruction for bigger cells is provided in the next section.

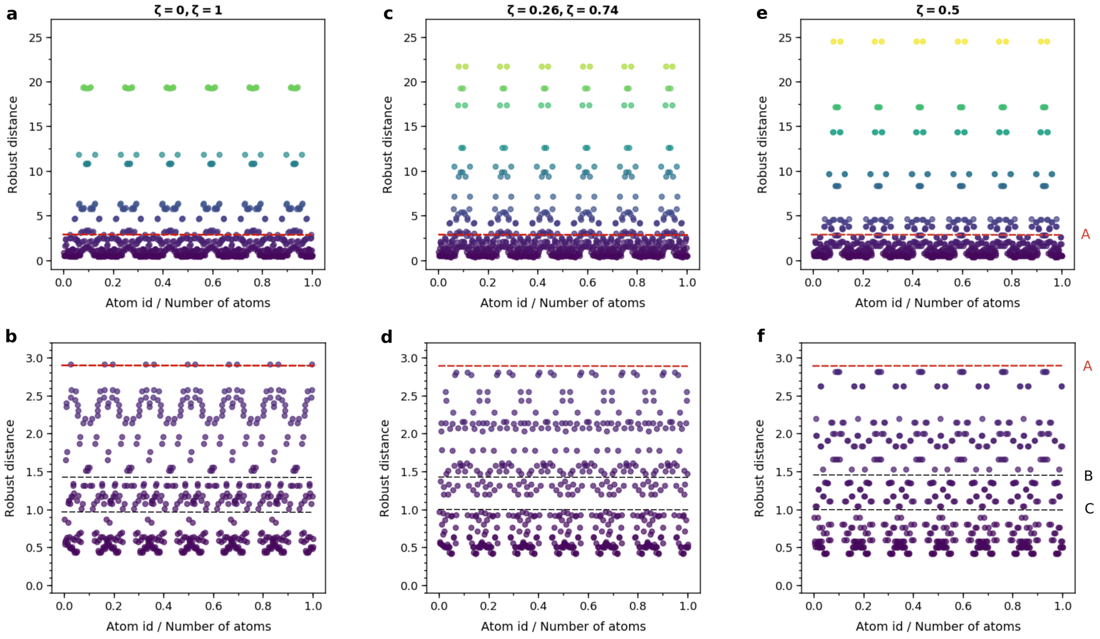

Supplementary Figure 5: Stratification of the small simulation cell with a dislocation dipole. The corresponding structures and the Peierls barriers are provided in Figure 5 c-d in the main text. MCD analysis is performed on the structural data represented using bispectrum SO(4) with $j_{max} = 4.0$ and $R_c = 5.0$. Each point on the plot represents an individual atom in the simulation box. Subplots (a,c,e) depict the full simulation cells with 810 atoms; subplots (b,d,f) depict the 582 atoms identified as bulk. Subplots (a,b); (c,d); (e,f) correspond to the snapshots with the reaction coordinate $\zeta = 0$, $\zeta = 1$; $\zeta = 0.26$, $\zeta = 0.74$ and $\zeta = 0.5$ along the NEB path in Figure 5c in the main text. The red dashed lines marked as A indicate the critical threshold $d_{RB} = 2.9$ identified by the MCD model. The black dashed lines at $d_{RB} = 1.45$ and $d_{RB} = 1.0$ marked as B and C in (b,d,f) indicate the possibilities for bulk stratification that these snapshots have in common.

It is worth emphasizing that an appropriate stratification of the bulk (Fig. 5b,d,f) is ensured by the training database of the MCD model. Bulk atoms represent inliers and their accurate description can be better obtained when using the training bulk structure from the relevant MD calculations. The training bulk structures with the noise generated be random displacements within the Gaussian distribution may not necessarily provide the results with sufficient accuracy to describe the subtle details of the displacement field produced by dislocations in the bulk structure.

It is also worth mentioning that the choice of atomic descriptors and their dimensionality has an impact on the computed distortion scores. Throughout this study, we employ bispectrum SO(4) [3]. This type of descriptors ensures a good radial and angular description of the structure, therefore they are commonly used for design of interatomic ML potentials [12, 33, 35]. We find that the

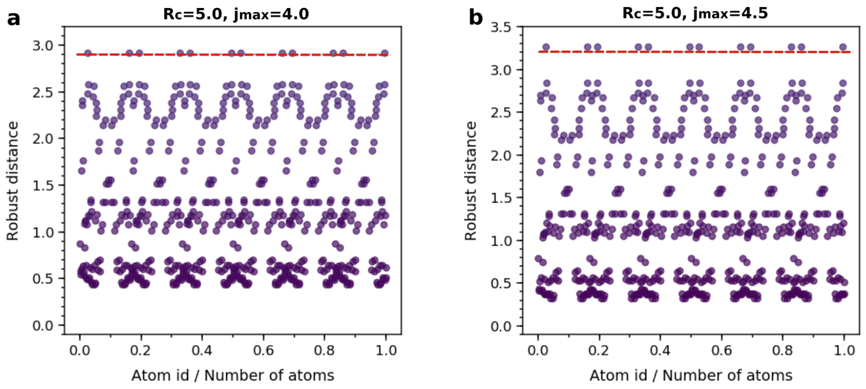

Supplementary Figure 6: Stratification of the bulk from small simulation cell with dislocation dipole (reaction coordinate $\zeta = 0$, $\zeta = 1$ in Fig. 5c in the main text) represented with bispectrum SO(4) with (a) $R_c = 5.0$ Å and $j_{max} = 4.0$ and (b) $R_c = 5.0$ Å and $j_{max} = 4.5$. The critical thresholds identified by the MCD model (a) $d_{RB} = 2.90$ and (b) $d_{RB} = 3.23$ are indicated with red dashed lines.

accurate description of the bulk structure (sufficient for the Peierls barrier reconstruction in bcc Fe) is provided by the $j_{max} = 4.0$ or $j_{max} = 4.5$ and $R_c = 5.0$. Figure 6 compares stratification of the bulk structure in the small simulation cell with the dislocation dipole obtained using the data representation with $j_{max} = 4.0$ or $j_{max} = 4.5$. The absolute values of the robust distances obtained with different j_{max} is different, but the overall pattern for the structure stratification remains similar (Fig. 6) and the subsets of atoms classified as inliers and outliers are the same.

Reconstruction of the Peierls barrier from mean force calculations in a big simulation cell

As in the case of a small dislocation dipole (main text, Fig. 5 c,d), the local definition of the dislocation core (Fig. 7b) is not sufficient to accurately reconstruct the Peierls barrier even if the dislocation cores are distant by more than 80 Å. Figure 8 illustrates the stratification of a big simulation cell with 20,250 atoms. From these stratification we define the three distortion scores: $d_{RB} = 3.23$ identified by the MCD model, $d_{RB} = 0.62$ and $d_{RB} = 0.55$ (Fig. 8b,d,f). The resulting structures and the Peierls barriers are provided in Figure 7.

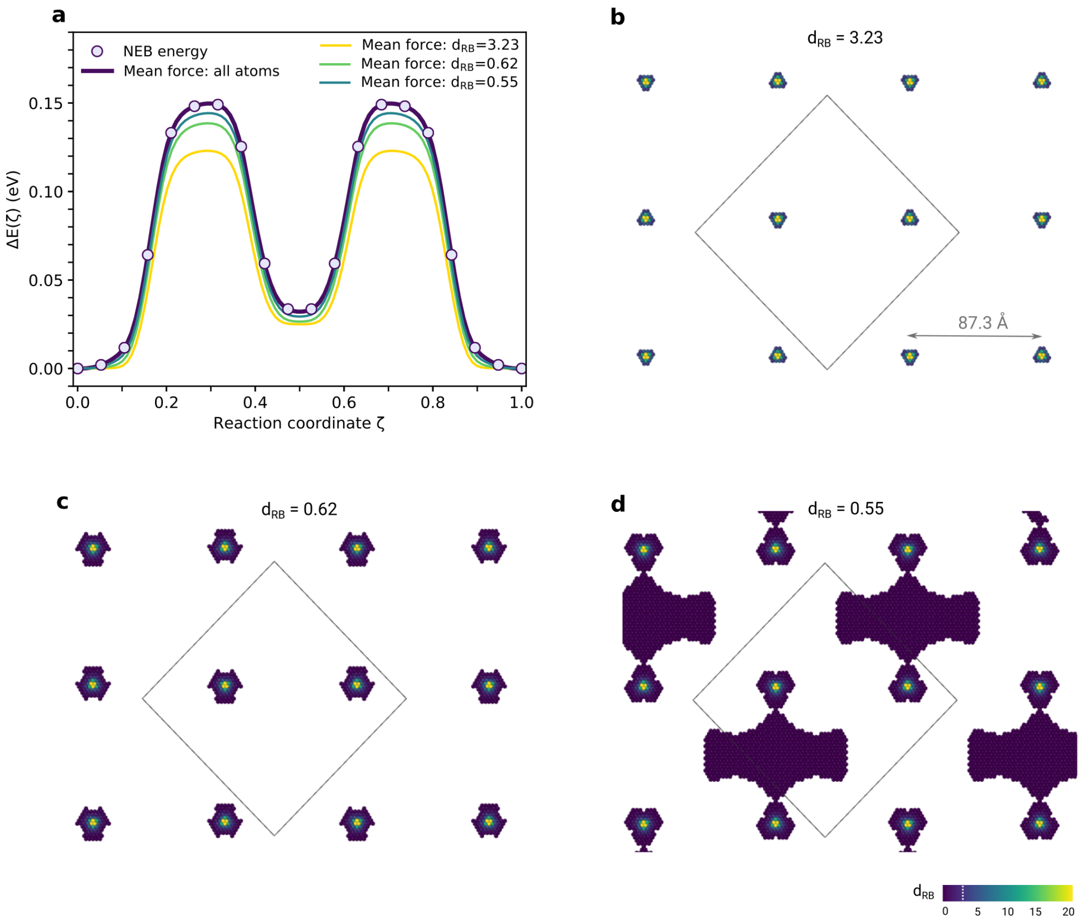

Supplementary Figure 7: (a) Energy profile of the screw $\frac{1}{2}\langle 111 \rangle$ dislocation dipole glide in $\{110\}$ plane in bcc Fe. Comparison of the total energy NEB calculations with the mean force integration over the confidence region v_{MCD} defined by different distortion score cutoff. The calculations are preformed using Ackland-Mendelev EAM potential [1]. (b-d) The stratified structures of the dislocations with different distortion score d_{RB} based on the robust MCD analysis. The critical threshold of the bulk structure is $d_{RB} = 3.23$. The atoms are coloured according to their distortion scores. The depicted structures are oriented along the $\langle 111 \rangle$ direction.

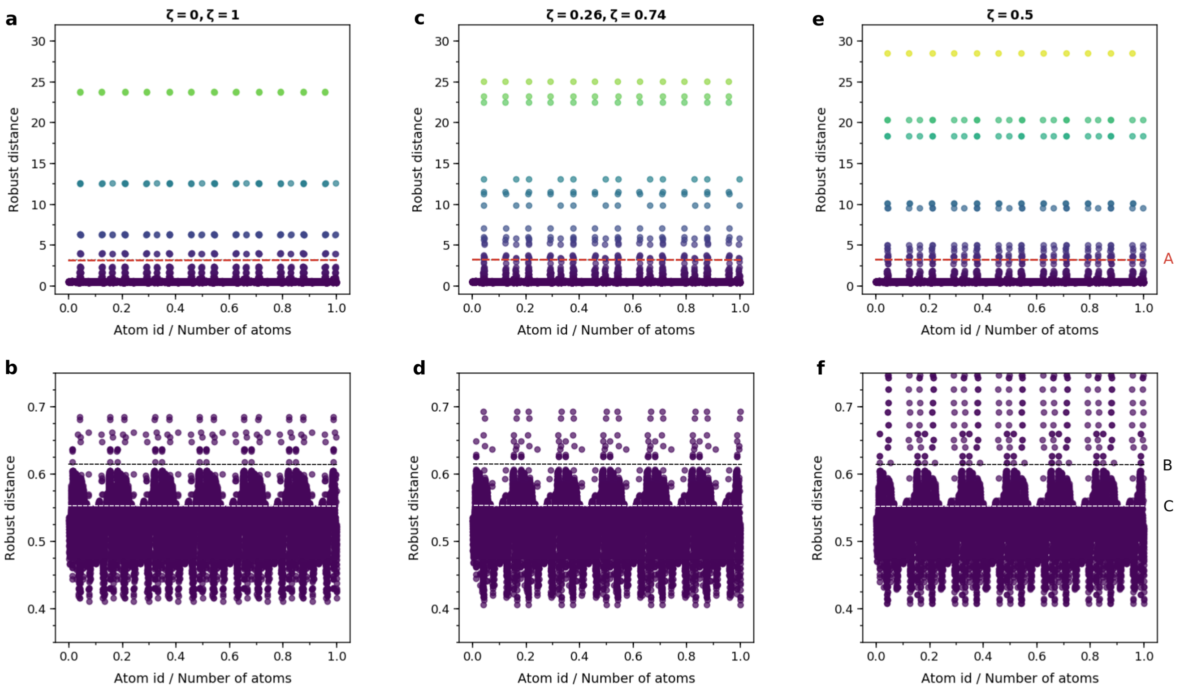

Supplementary Figure 8: Stratification of the big simulation cell with dislocation dipole. The corresponding structures and Peierls barriers are provided in Figure 7. MCD analysis is performed on the structural data represented using bispectrum SO(4) with $j_{max} = 4.5$ and $R_c = 5.0$. Each point on the plot represents an individual atom in the simulation box. Subplots (a,c,e) depict the full simulation cells with 20,250 atoms; subplots (b,d,f) depict the 20,022 atoms identified as bulk. Subplots (a,b); (c,d); (e,f) correspond to the snapshots with the reaction coordinate $\zeta = 0$, $\zeta = 1$; $\zeta = 0.26$, $\zeta = 0.74$ and $\zeta = 0.5$ along the NEB path in Figure 7a. The red dashed lines marked as A indicate the critical threshold $d_{RB} = 3.23$ between inliers and outliers provided by the MCD model. The black dashed lines at $d_{RB} = 0.62$ and $d_{RB} = 0.55$ marked as B and C in (b,d,f) indicate the possibilities for bulk stratification.

When taking into account only atoms-outliers ($d_{RB} > 3.23$), the migration barrier is underestimated by more than 20 %. Including the relevant bulk atoms with distortion scores greater than 0.62 (Fig. 7c) and 0.55 (Fig. 7d) and thereby taking into account the elastic interaction of the cores significantly improves the results.

IV. SUPPLEMENTARY NOTE 4: PERFORMANCE OF THE GAP POTENTIAL FOR MODELLING RADIATION INDUCED DEFECTS IN BCC IRON

Radiation induced atomic displacements mainly occur in the form of point defects, e.g., self-interstitial atoms (SIAs), vacancies, Frenkel pairs. These defects may eventually recombine and annihilate or regroup and form larger defects like 2D and 3D clusters, dislocation loops, stacking faults, etc. The defects adopt different morphologies, which further define their size-dependent mobility and stability with respect to each other. In bcc Fe, low energy $\langle 111\rangle$ and $\langle 110\rangle$ loops are characterised by a high mobility (with migration energy barriers in the order of meV and 0.3 eV, respectively), while the higher energy $\langle 100\rangle$ loops are immobile (migration energy barriers in the order of few eV). The C15 SIA clusters have a 3D crystallographic structure, which makes them intrinsically immobile. These complex and highly symmetric defects act as sinks for other small defects. By absorbing neighbouring SIAs, C15 clusters grow larger and, after accumulating more than 50 point defects, they may eventually dissociate into $\langle 111\rangle$ and $\langle 100\rangle$ loops [2]. Overall, the energy landscape of defects in bcc Fe is extremely complex and its accurate description at the atomic scale requires using appropriate force-field models that provide a correct description of atomic systems beyond the equilibrium conditions.

Below we report the results of the formation and migration energy calculations using GAP potential [9] and compare them with those from the DFT calculations and from the two EAM potentials commonly used for modelling defects in bcc Fe: AM04 [1] and MA07 [18, 21].

Self-interstitial atoms and their clusters

In contrast to the equilibrium conditions where vacancies represent the dominant part of self-defects, self-interstitial atoms (SIAs) occur in the same amount as vacancies under irradiation. Mobility and relative stability of SIAs in bcc Fe strongly depend on their geometry.

For single SIAs, resistivity recovery experiments [10, 32], DFT calculations [11, 34], EAM [18, 34] and the tested GAP potential [9] consistently indicate that the dumbbells with $\langle 110\rangle$ orientation are the most stable. These dumbbells can migrate from their initial position to the next site via several different jump mechanisms [11]. Figure 9 illustrates the most important of them. The computed migration energy barriers for each of these mechanisms are summarised in Table I. For all the mechanisms (Fig. 9), the GAP potential performs better than any EAM potential and provides the energy barriers very close (within 10% difference) to the DFT values. Although the

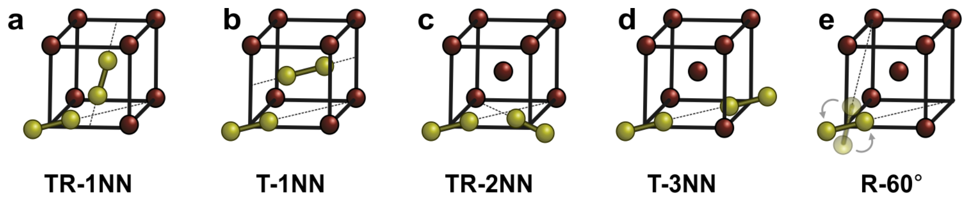

Supplementary Figure 9: Migration mechanisms of ⟨110⟩ SIA dumbbells in bcc Fe: (a) translation and rotation to the first nearest neighbour position; (b) translation to the first nearest neighbour position; (c) translation and rotation to the second nearest neighbour site; (d) translation to the third nearest neighbour position; (e) 60° on-site rotation. The corresponding migration energy barriers are reported in Table I

exact trajectories of the jump mechanisms were not explicitly included into the training database of the GAP potential [9], it can accurately compute the migration barriers for all the five mechanisms. Such a good performance of the potential likely results from the rich variety of single SIA atomic environments in the training data set [9].

Supplementary Table I: Migration energy barriers ΔE_m of single ⟨110⟩ SIA dumbbells computed for five different jump mechanisms in bcc Fe using GAP [9] and EAM potentials AM04 [1], MA07 [18, 21]. The results are compared with the DFT values from Ref. [11]. The listed mechanisms correspond to those illustrated in Fig. 9

Mechanism	Energy barrier ΔE_m, eV			
	AM04	MA07	GAP	DFT [11]
1: TR-1NN	0.30	0.27	0.32	0.34
2: T-1NN	0.43	0.62	0.70	0.78
3: TR-2NN	0.62	0.61	0.54	0.50
4: T-3NN	0.67	1.21	1.32	1.18
5: $R-60°$	0.41	0.49	0.64	0.56

With increasing size, SIA clusters change their relative stability. For 2D loops with more than 4 SIAs, the ⟨111⟩ clusters become more stable than the ⟨110⟩ family [34]. Further we test the performance of GAP to correctly reproduce size-dependent stability of SIA clusters, including 2D loops (Fig. 10a) and more complex 3D C15 clusters (Fig. 10b).

The qualitative predictions of the relative loop stability and the crossover between the ⟨111⟩ and ⟨110⟩ families (Fig. 10a) is slightly overestimated with respect to the DFT calculations [34]. Overall, for the small 2D loops, GAP leads to the results very similar to those from the MA07 potential and predicts the transition between the ⟨111⟩ and ⟨110⟩ loops after accumulation of 5

Supplementary Table II: Migration energies ΔE_m of the most stable small SIAs (I_{1-3}) and vacancy (V_{1-3}) clusters (from 1 to 3 defects) with the lowest migration energy computed using GAP [9] and EAM potentials AM04 [1], MA07 [18, 21]. The results are compared with the DFT values from Ref. [10].

	SIA ΔE_m, eV			Vacancy ΔE_m, eV		
	I_1	I_2	I_3	V_1	V_2	V_3
AM04	0.30	0.33	0.32	0.64	0.71	0.55
MA07	0.27	0.32	0.34	0.68	0.71	0.43
GAP	0.32	0.37	0.31	0.67	0.54	0.14
DFT [10]	0.34	0.43	0.43	0.67	0.62	0.35

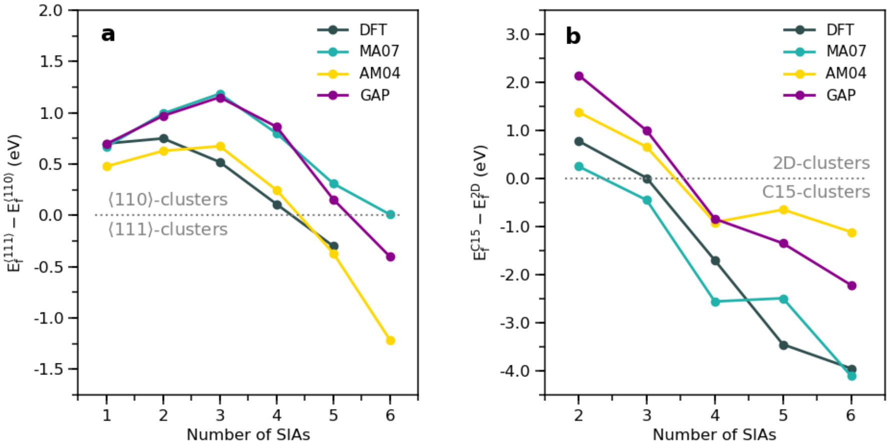

Supplementary Figure 10: Size dependent stability of (a) ⟨111⟩ small clusters with respect to ⟨110⟩ loops; (b) the C15 clusters with respect to the lowest energy 2D clusters configurations from (a). The DFT values for computing the difference in formation energies (a) $\Delta E_f = E_f^{\langle 111\rangle} - E_f^{\langle 110\rangle}$ are taken from Ref. [34]; (b) $\Delta E_f = E_f^{C15} - E_f^{2D}$ are taken from Ref. [20].

SIAs, which is slightly bigger than the cluster sizes obtained by DFT and AM04 potential. The energy difference $\Delta E_f = E_f^{\langle 111\rangle} - E_f^{\langle 110\rangle}$ provided by the GAP potential for the SIA loops that are built by more than 3 dumbbells has more than 50% error with respect to the DFT calculations [34]. Thus, for the size-dependent formation energies of 2D SIA clusters, the GAP potential exhibits a lower accuracy compared to its performance for single SIAs.

The relative stability of the C15 clusters with respect to the 2D loops is illustrated in (Fig. 10b). As was previously shown in Refs. [2, 6, 21], AM04 potential poorly reproduces the energy landscape of C15 compared to DFT calculations. This results from the fact that the small $I_{2,3}^{C15}$ clusters are less stable than the dumbbell configurations and the difference between the formation

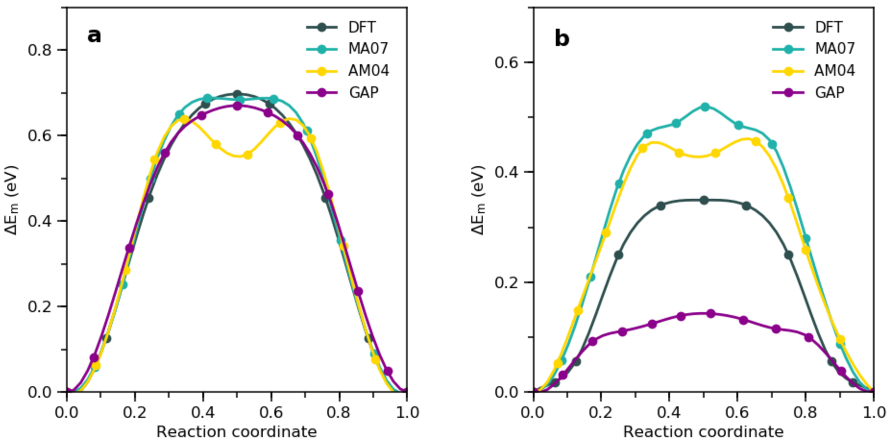

Supplementary Figure 11: Migration energy barriers ΔE_m (a) of mono-vacancy V_1 and (b) of tri-vacancy V_3 cluster. The DFT curves are computed with VASP using PBE pseudopotential with $3d^7 4s^1$ electrons.

energy of C15 and ⟨111⟩ loops is very small. The second EAM potential, MA07, performs much closer to the DFT data [20]. In contrast to the 2D loops where the GAP provides qualitatively good results, the tested ML potential exhibits a limited transferability for the C15 clusters and provides results close to those from AM04 in Fig. 10b. For the small size $I_{2,3}^{C15}$ clusters, the GAP potential provides the formation energies that are much higher than those of dumbbell configurations. The predicted formation energy of I_2^{C15} is *c.a.* 2.5 eV higher than that of ⟨110⟩ dumbbells, yielding an impossible formation of C15 in Fe. Moreover, for larger clusters, the GAP suggests that the C15 clusters are less stable compared to the ⟨111⟩ loops. These results are not consistent with the DFT calculations [2, 20].

It is interesting to note that both MA07 and GAP were not fitted to compute C15 (i.e., the C15 clusters were not explicitly included in the potential databases). However, the fitting databases of both potentials contained the so-called nonparallel (NP) di-interstitial configuration I_2^{NP}. The I_2^{NP} configuration represents an elementary building block required to create a C15 cluster [2, 21]. Thus, in the case of MA07 EAM potential (which is based on a relatively simple physical concept), including such a brick was sufficient to reconstruct more complex structural defect, while in the case of GAP (which relies on statistical methods), the outcome is not the same. As a possible solution, one may consider including more complex defect structures into the training databases of ML potentials in order to enrich the variety of atomic environments known by the model.

Vacancies and their clusters

In the original publication of the GAP potential [9], the authors provide the calculations of the formation and binding energies of mono- di- and tri-vacancies. These properties were directly incorporated into the training dataset. Hence, the performance of the potential on these properties is excellent and closely consistent with the reference DFT calculations [9]. In this work we do not intend to reaffirm and discuss these calculations.

The migration energy barriers of mono-vacancies to the first, second, and third nearest neighbour site were also reported by Dragoni et al. [9]. It is worth emphasizing that the migration energy barriers of mono-vacancies computed with GAP are remarkably accurate compared to those provided by existing EAM potentials (Table II). Moreover, the energy barrier of monovacancy migration from GAP calculations exhibits a clear saddle point (Fig. 11a), while it is not the case for the EAM potentials.

We further test GAP to compute migration energy barriers of di- and tri-vacancy clusters. For the small $V_{2,3}$ clusters, GAP also provides a single saddle-point curves, however the computed energy barriers are notably lower than the corresponding DFT values (Table II). Thus, for the di-vacancies V_2, the computed energy barrier is 25% lower than the DFT migration energy, while for the tri-vacancies V_3, the error reaches 60% (Table II, Fig. 11b). Such an error in migration barriers will have a strong impact on the predictions of defect kinetics under irradiation and interpretation of processes during resistivity recovery experiments. The stage IV will be strongly impacted by the fast diffusion of the vacancies clusters V_n with $n > 2$ [10, 32] affecting the prediction of the size and the density of vacancy clusters at temperatures higher than 300 K.

Methods

Atomistic calculations with semi-empirical and ML force fields are performed in LAMMPS [24] using the AM04 [1], MA07 [18, 21] EAM potentials and the GAP potential [9] for Fe. The calculations are carried out at the constant volume of the $8 \times 8 \times 8$ bcc simulation cells with a_0=2.834 Å and a_0=2.8553 Å for GAP and EAM (AM04 and MA07) potentials, respectively. The atomic arrays with SIAs and vacancies contain in the order of 1016-1032 atoms, dependently on the defect type. Molecular static energy minimization is performed using a conjugate gradient with 10^{-5} eV/Å (1.602×10^{-14} N) convergence criterion for forces. The migration and transition barriers (Tables I, II; Fig. 11) are obtained from the NEB calculations performed via fire damped dynam-

ics. The minimum energy path (MEP) is sampled with 12 points for the migration of SIA and vacancies. The spring constant for parallel nudging force is set to 15.0 eV/Å.

Ab initio calculations of the mono- and tri- vacancy migration barriers (Fig. 11) are performed for the simulation cells with 127 and 125 atoms at the constant volume of $4 \times 4 \times 4$ bcc simulation cells with a_0=2.834 Å. The simulations are carried out in VASP [17] using PAW pseudopotential with GGA-PBE functional that accounts for 8 valence electrons $3d^7 4s^1$. The plane-wave cutoff energy is set to 500 eV. The Brillouin zone is sampled with the Monkhorst-Pack scheme using $4 \times 4 \times 4$ k-point grid with 0.5 shift. The MEP of vacancy clusters migration is sampled with 9 points.

SUPPLEMENTARY REFERENCES

[1] G. J. Ackland, M. I. Mendelev, D. J. Srolovitz, S. Han, and A. V. Barashev. Development of an interatomic potential for phosphorus impurities in α-iron. *J. Phys. Condens. Matter*, 16:2629, 2004.

[2] R. Alexander, M.-C. Marinica, L. Proville, F. Willaime, K. Arakawa, M. R. Gilbert, and S. L. Dudarev. *Ab initio* scaling laws for the formation energy of nanosized interstitial defect clusters in iron, tungsten, and vanadium. *Phys. Rev. B*, 94:024103, 2016.

[3] A. P. Bartók, R. Kondor, and G. Csányi. On representing chemical environments. *Phys. Rev. B*, 87:184115, 2013.

[4] J. Behler. Atom-centered symmetry functions for constructing high-dimensional neural network potentials. *J. Chem. Phys.*, 134(7):074106, 2011.

[5] U. Bhardwaj, A. E. Sand, and M. Warrier. Classification of clusters in collision cascades. *Computational Materials Science*, 172:109364, 2020.

[6] J. Byggmastar, F. Granberg, A. E. Sand, A. Pirttikoski, R. Alexander, M.-C. Marinica, and K. Nordlund. Collision cascades overlapping with self-interstitial defect clusters in Fe and W. *J. Phys.: Condens. Matter*, 31(24):245402, 2019.

[7] M. Caturla, N. Soneda, E. Alonso, B. Wirth, T. Dìaz de la Rubia, and J. Perlado. Comparative study of radiation damage accumulation in Cu and Fe. *Journal of Nuclear Materials*, 276(1):13 – 21, 2000.

[8] E. Cubuk, S. S. Schoenholz, J. M. Rieser, B. D. Malone, J. Rottler, D. J. Durian, E. Kaxiras, and A. J. Liu. Identifying structural flow defects in disordered solids using machine-learning methods. *Phys. Rev. Lett.*, 114:108001, 2015.

[9] D. Dragoni, T. D. Daff, G. Csányi, and N. Marzari. Achieving DFT accuracy with a machine-learning

interatomic potential: Thermomechanics and defects in bcc ferromagnetic iron. *Phys. Rev. Materials*, 2:013808, 2018.

[10] C.-C. Fu, J. D. Torre, F. Willaime, J.-L. Bocquet, and A. Barbu. Multiscale modelling of defect kinetics in irradiated iron. *Nature Materials*, 4:68–74, 2005.

[11] C.-C. Fu, F. Willaime, and P. Ordejón. Stability and mobility of mono- and di-interstitials in α-Fe. *Phys. Rev. Lett.*, 92:175503, 2004.

[12] A. M. Goryaeva, J.-B. Maillet, and M.-C. Marinica. Towards better efficiency of interatomic linear machine learning potentials. *Computational Materials Science*, 166:200 – 209, 2019.

[13] M. Hubert and M. Debruyne. Minimum covariance determinant. *Wiley Interdisciplinary Reviews: Computational Statistics*, 2:36–43, 2010.

[14] M. Hubert, M. Debruyne, and P. J. Rousseeuw. Minimum covariance determinant and extensions. *Wiley Interdisciplinary Reviews: Computational Statistics*, 10:e1421, 2018.

[15] K. W. Ingle, R. C. Perrin, and H. R. Schober. Interstitial cluster in FCC metals. *J. Phys. F: Met. Phys.*, 11(6):1161, 1981.

[16] M. I. Jordan and R. A. Jacobs. Hierachical mixtures of experts and the em algorithm. *Neural Computation*, 6:182, 1994.

[17] G. Kresse and J. Furthmüller. Efficient iterative schemes for *ab initio* total energy calculations using a plane-wave basis set. *Phys. Rev. B*, 54:11169–11186, 1996.

[18] L. Malerba, M. M.-C, N. Anento, C. Björkas, H. Nguyen, C. Domain, F. Djurabekova, P. Olsson, K. Nordlund, A. Serra, D. Terentyev, F. Willaime, and C.-S. Becquart. Comparison of empirical interatomic potentials for iron applied to radiation damage studies. *J. Nucl. Mater*, 406:19–38, 2010.

[19] J. M. Marin, K. Mengersen, and C. P. Robert. *Bayesian modelling and inference on mixtures of distributions*. Elsevier, 2011.

[20] M.-C. Marinica, L. Ventelon, M. R. Gilbert, L. Proville, S. L. Dudarev, J. Marian, G. Bencteux, and F. Willaime. Interatomic potentials for modelling radiation defects and dislocations in tungsten. *J. Phys. Condens. Matter*, 25(39):395502, 2013.

[21] M.-C. Marinica, F. Willaime, and J.-P. Crocombette. Irradiation-induced formation of nanocrystallites with C15 laves phase structure in bcc iron. *Phys. Rev. Lett.*, 108:025501, 2012.

[22] M. Mendelev, M. Kramer, C. Becker, and M. Asta. Analysis of semi-empirical interatomic potentials appropriate for simulation of crystalline and liquid Al and Cu. *Philosophical Magazine*, 88(12):1723–1750, 2008.

[23] K. Nordlund, S. Zinkle, A. Sand, F. Granberg, R. Averback, R. Stoller, T. Suzudo, T. Suzudo, L. Malerba, F. Banhart, W. Weber, F. Willaime, S. Dudarev, and D. Simeone. Improving atomic displacement and replacement calculations with physically realistic damage models. *Nature Communications*, 9:1084, 2018.

[24] S. Plimpton. Fast parallel algorithms for short-range molecular dynamics. *Journal of Computational Physics*, 117(1):1 – 19, 1995.

[25] P. J. Rousseeuw and M. Hubert. Anomaly detection by robust statistics. *Wiley Interdisciplinary Reviews: Data Mining and Knowledge Discovery*, 8(2):e1236, 2018.

[26] A. E. Sand, S. L. Dudarev, and K. Nordlund. High-energy collision cascades in tungsten: Dislocation loops structure and clustering scaling laws. *EPL (Europhysics Letters)*, 103(4):46003, 2013.

[27] S. S. Schoenholz, E. D. Cubuk, E. Kaxiras, and A. J. Liu. Relationship between local structure and relaxation in out-of-equilibrium glassy systems. *Proceedings of the National Academy of Sciences*, 114(2):263–267, 2017.

[28] S. S. Schoenholz, E. D. Cubuk, D. M. Sussman, E. Kaxiras, and A. J. Liu. A structural approach to relaxation in glassy liquids. *Nature Physics*, 12:469–472, 2016.

[29] B. Schölkopf, J. C. Platt, J. Shawe-Taylor, A. J. Smola, and R. C. Williamson. Estimating the support of a high-dimensional distribution. *Neural Computation*, 13(7):1443–1471, 2001.

[30] B. Schölkopf, R. Williamson, A. Smola, J. Shawe-Taylor, and J. Platt. Support vector method for novelty detection. In *Advances in Neural Information Processing Systems 12*, pages 582–588. MIT Press, 2000.

[31] T. A. Sharp, S. L. Thomas, E. D. Cubuk, S. S. Schoenholz, D. J. Srolovitz, and A. J. Liu. Machine learning determination of atomic dynamics at grain boundaries. *Proceedings of the National Academy of Sciences*, 115(43):10943–10947, 2018.

[32] S. Takaki, J. Fuss, H. Kuglers, U. Dedek, and H. Schultz. The resistivity recovery of high purity and carbon doped iron following low temperature electron irradiation. *Radiation Effects*, 79(1-4):87–122, 1983.

[33] A. Thompson, L. Swiler, C. Trott, S. Foiles, and G. Tucker. Spectral neighbor analysis method for automated generation of quantum-accurate interatomic potentials. *Journal of Computational Physics*, 285:316 – 330, 2015.

[34] F. Willaime, C. Fu, M. Marinica, and J. D. Torre. Stability and mobility of self-interstitials and small interstitial clusters in α-iron: *ab initio* and empirical potential calculations. *Nuclear Instruments and Methods in Physics Research Section B: Beam Interactions with Materials and Atoms*, 228(1):92 – 99, 2005.

[35] M. A. Wood and A. P. Thompson. Quantum-accurate molecular dynamics potential for tungsten. *arXiv:1702.07042v1 [physics.comp-ph]*, 2017.

[36] X. Yi, A. E. Sand, D. R. Mason, M. A. Kirk, S. G. Roberts, K. Nordlund, and S. L. Dudarev. Direct observation of size scaling and elastic interaction between nano-scale defects in collision cascades. *EPL (Europhysics Letters)*, 110(3):36001, 2015.
